# Effects of endocrine disruptors on thyroid function: consequences of fetal exposure

**DOI:** 10.55730/1300-0144.6122

**Published:** 2025-10-26

**Authors:** Esra ERASLAN AYDEMİR, Mustafa ŞAHİN

**Affiliations:** Department of Endocrinology and Metabolism, Faculty of Medicine, Ankara University, Ankara, Turkiye

**Keywords:** Endocrine disruptors, thyroid dysfunction, fetal development, neurogenesis, environmental exposure

## Abstract

Fetal development depends on complex maternal–fetal–placental interactions, with thyroid hormones playing a vital role in regulating growth and neurogenesis. Exposure to endocrine-disrupting chemicals (EDCs) during pregnancy has emerged as a significant risk factor for thyroid dysfunction and its associated developmental and cognitive disorders. EDCs, including bisphenol A (BPA), polychlorinated biphenyls (PCBs), polybrominated diphenyl ethers (PBDEs), perfluoroalkyl substances, pesticides, and heavy metals, disrupt thyroid hormone synthesis, secretion, and metabolism.

Mechanisms involve receptor binding, disruption of the hypothalamic–pituitary–thyroid axis, and inhibition of thyroid peroxidase activity. BPA exposure, for instance, reduces free and total T4 levels and interferes with deiodinase activity. Similarly, PCBs and PBDEs are associated with lower thyroxine concentrations and long-term behavioral abnormalities in offspring. Pesticides and heavy metals exacerbate thyroid dysfunction by interfering with hormone synthesis and receptor interactions. Genetic predisposition, iodine deficiency, and autoimmune conditions further increase susceptibility to EDC-related thyroid disorders.

Considering the heightened vulnerability of early pregnancy and the widespread environmental presence of EDCs, reducing exposure and implementing regulatory measures are essential to mitigate their adverse effects on maternal and fetal thyroid health. Future research should prioritize elucidating the mechanisms of EDC-induced thyroid dysfunction and developing interventions to protect at-risk populations.

## 1. Introduction

Fetal development is a process regulated by complex mechanisms involving fetal–placental and maternal factors. Adequate maternal intake of nutrients–carbohydrates, proteins, lipids, vitamins, and minerals–is essential for fetal growth, which largely depends on the mother’s endocrine status [1]. Recently, researchers have investigated the effects of ubiquitous environmental chemicals that adversely affect both maternal and fetal health [2]. Among these substances, endocrine-disrupting chemicals (EDCs) exert significant effects on the pregnant woman, placenta, and fetus. Samples from pregnant women have revealed the presence of organochlorine pesticides, phenols, polychlorinated biphenyls, polybrominated diphenyl ethers, phthalates, and polycyclic aromatic hydrocarbons in 99%–100% of cases [3]. Similarly, placental evaluations have detected various EDCs—originating from personal care products, pesticides, polybrominated diphenyl ethers, and bisphenol A—which are associated with fetal growth restriction, neurodevelopmental disorders, and thyroid dysfunction [4].

Given the critical role of thyroid hormones in growth and development, exposure to EDCs during fetal and neonatal periods may impair thyroid function and neural development. EDCs can disrupt thyroid function by binding to thyroid hormone receptors, altering triiodothyronine (T3) and thyroxine (T4) secretion, synthesis, and metabolism, thereby leading to neurodevelopmental impairments. Developmental exposure is particularly critical because thyroid dysfunction can profoundly impair neurogenesis [5]. Experimental studies have demonstrated that prenatal exposure to EDCs disrupts thyroid function, resulting in cognitive and behavioral abnormalities. Evidence from numerous epidemiological and experimental studies indicates that early pregnancy is the most vulnerable period to EDC exposure, and even low-level exposure can result in significant adverse outcomes [6].

EDCs occur as mixtures in real-world environments and exert additive or synergistic effects on the thyroid system through a “cocktail effect”. During pregnancy, combinations of various EDCs—including per- and polyfluoroalkyl substances (PFAS), BPA, phthalates, and pesticides—even at low doses, interfere with thyroid hormone synthesis, transport, and metabolism through multiple mechanisms, producing additive neurodevelopmental effects that exceed those expected from individual chemical exposures [7].

Studies from different countries have demonstrated that prenatal exposure to EDCs leads to adverse neurodevelopmental outcomes in children, including impaired gross motor development, reduced IQ scores, learning disabilities, and attention deficit hyperactivity disorder (ADHD). Notably, male fetuses appear to be more susceptible to EDC-induced neurodevelopmental effects, with studies reporting greater IQ deficits and behavioral problems in boys than in girls [7,8].

## 2. Major endocrine disruptors and their fetal effects

Bisphenol A (BPA), a chemical widely used in plastic manufacturing, is commonly found in water and milk bottles, feeding bottles, electronic devices, and automotive materials. Exposure to BPA during early pregnancy (approximately 6–14 weeks) has been shown to impair thyroid function and alter deiodinase activity. BPA exposure is associated with reduced free and total T4/T3 ratios and decreased total T4 levels [9]. Another study reported an inverse correlation between maternal urinary BPA levels and neonatal serum thyroid-stimulating hormone (TSH) concentrations, but not with free T3 and T4 levels [10]. Prenatal BPA exposure has been shown to induce neurobehavioral alterations later in life by disrupting thyroid hormone homeostasis [11].

A study conducted at Johns Hopkins Hospital by Herbstman et al. demonstrated that prenatal exposure to environmental EDCs—such as polychlorinated biphenyls (PCBs) and polybrominated diphenyl ethers (PBDEs)—results in decreased total and free thyroxine levels in infants [12]. A cohort study in China found that low-level prenatal exposure to PBDEs was associated with decreased thyroid hormone levels in cord plasma and long-term behavioral abnormalities in children [13].

Per- and polyfluoroalkyl substances (PFAS) are widely used chemicals that bioaccumulate in the environment and in humans over time. They are present in numerous everyday materials, including fire-extinguishing foams, textiles, furniture, carpets, cosmetic products, and nonstick coatings [14,15]. Approximately 4000 perfluorinated compounds are currently in production, with perfluorooctanoic acid and perfluorooctanesulfonic acid detected in amniotic fluid and recognized for their antithyroid effects [16].

Perchlorate, nitrate, and thiocyanate anions inhibit the sodium–iodide symporter, with perchlorate being approximately 20 times more potent than thiocyanate and over 500 times more potent than nitrate. Detected in amniotic fluid, perchlorate is ingested via drinking water and exposure to active or passive smoking, thereby affecting maternal thyroid hormone levels and fetal thyroid gland development [17]. Studies have shown that maternal perchlorate exposure is associated with impaired cognitive function in newborns [18].

## 3. The impact of endocrine disruptors on thyroid hormone synthesis and function

Recent studies have demonstrated that exposure to EDCs can alter normal thyroid hormone levels, representing a significant exogenous risk factor for the onset and progression of thyroid disorders. EDCs can induce thyroid dysfunction by directly damaging thyroid tissue, competitively binding to thyroid hormone receptors, disrupting the hypothalamic–pituitary–thyroid axis, dysregulating thyroid disease–related genes such as the proto-oncogene KRAS and the tumor suppressor phosphatase and tensin homolog, and activating intracellular signaling pathways including extracellular signal–regulated kinase and protein kinase B [19]. Continuous exposure to EDCs may cause compensatory hyperplasia and hypertrophy of thyroid tissue, ultimately contributing to thyroid pathology. The impact of endocrine disruptors on thyroid function is more pronounced in regions with iodine deficiency, in individuals with preexisting thyroid autoimmunity, and in those carrying mutations in deiodinase genes. EDCs may also disrupt thyroid hormone synthesis and function by triggering autoimmune thyroid disorders. In a study by Sur et al., an association was identified between bisphenol A exposure and Hashimoto’s thyroiditis [20].

### 3.1. Effects of some important endocrine disruptors on thyroid hormone synthesis

BPA can induce thyroid dysfunction through multiple mechanisms, including direct cytotoxicity to pituitary and thyroid cell lines, as well as antagonistic effects on thyroid hormone receptors that modify transcriptional activity [21]. Owing to its structural similarity to thyroid hormones, BPA competitively inhibits T3 binding to its receptor, thereby impeding normal transcriptional activity [22]. BPA also binds to serum transport proteins of T3 and T4, disrupting normal thyroid hormone transport and function [21].

The prevalence and incidence of thyroid disorders have increased in regions of Spain with intensive pesticide use [23]. In southern Brazil, significant pesticide exposure among soybean farmers has been associated with lower serum TSH levels and elevated total T3 and free T4 concentrations [24]. Certain pesticides and fungicides inhibit thyroid peroxidase activity, thereby altering thyroid hormone synthesis and circulating levels. Intensive pesticide use in agricultural regions poses a potential risk for thyroid dysfunction.

Workers exposed to cadmium exhibit a direct correlation between urinary cadmium concentrations and serum TSH levels, and an inverse correlation with free T3 and T4 levels [25]. Mercury inhibits the enzyme 5-deiodinase, which catalyzes the conversion of T4 to T3, and occupational exposure to mercury vapor has been associated with reduced serum free T3 concentrations.

Phthalates are synthetic compounds used as plasticizers in a wide range of products, including food packaging, bottles, toys, paints, and cosmetics. Research has indicated that the antithyroid activity of phthalates is mediated by a gram-negative bacterium exhibiting thyroid peroxidase–like activity, and these compounds are classified as “possible human carcinogens” [26]. Some studies have reported an inverse association between urinary phthalate concentrations and serum total and free T4 levels, whereas others have demonstrated a positive correlation with serum TSH levels [27]. Phthalates can act as both thyroid hormone receptor agonists and antagonists. Exposure to di(2-ethylhexyl) phthalate inhibits thyroid hormone biosynthesis, decreases sodium–iodide symporter expression, alters deiodinase and transthyretin levels, and accelerates the progression of preexisting thyroid disorders [28].

PBDEs, used as flame retardants, have been associated with hypothyroidism following environmental exposure [29]. Perchlorate and thiocyanate anions inhibit the sodium–iodide symporter, thereby reducing iodine uptake into the thyroid and impairing thyroid hormone synthesis [30]. Table presents the different mechanisms of action of various endocrine-disrupting agents on thyroid hormone synthesis and function [31].

## 4. Preventive strategies and lifestyle recommendations

Iodine deficiency is a critical factor that amplifies the adverse effects of EDCs on thyroid function. Adequate maternal iodine intake (approximately 250 μg/day during pregnancy) may offer partial protection against the thyroid hormone synthesis–disrupting effects of EDCs, particularly by mitigating the inhibitory impact of sodium–iodide symporter antagonists such as perchlorate, thiocyanate, and nitrate. However, excessive iodine intake may also lead to thyroid dysfunction; therefore, iodine supplementation should be administered under medical supervision following an assessment of regional iodine status [32].

Evidence-based lifestyle recommendations to minimize EDC exposure during pregnancy include the following: using glass or stainless-steel food containers instead of plastic; avoiding the use of plastic in microwave ovens (to reduce BPA and phthalate exposure); consuming organic foods (to limit pesticide exposure); choosing fragrance-free and phthalate-free personal care products; using HEPA-filtered air purifiers at home (to reduce airborne EDCs); and avoiding processed or packaged foods. Additionally, pregnant women should minimize the use of nonstick cookware (to reduce PFAS exposure), choose fresh or frozen alternatives to canned foods, and avoid handling thermal paper receipts. These simple preventive measures can substantially reduce the total EDC burden and help preserve fetal thyroid function [33].

## 5. Conclusion

EDCs represent a critical public health concern by impairing thyroid hormone synthesis and function, thereby jeopardizing fetal neurodevelopment. Prenatal exposure to EDC mixtures leads to irreversible neurocognitive impairment and measureable IQ reduction. Although optimal iodine supplementation and evidence-based lifestyle modifications can mitigate exposure, individual measures alone remain insufficient. Systematic public health interventions—including class-based bans on EDCs, routine screening during pregnancy, and education of healthcare professionals—are urgently required. Considering the transgenerational consequences, the actions taken today will determine the health trajectory of future generations. Therefore, the rapid translation of scientific evidence into policy and clinical practice is imperative.

## Figures and Tables

**Table:** Various endocrine-disrupting agents and their mechanisms of action on thyroid hormones.

Mechanism	Endocrine disruptors
**Induction of hepatic thyroid hormone metabolism**	Dioxins, PBDEs, pesticides
**Thyroperoxidase inhibition**	Isoflavones, pesticides, PFAS
**Inhibition of sodium–iodide symporter**	Nitrate, perchlorate, PFAS, thiocyanate
**Activation or inhibition of thyroid hormone receptor**	BPA, PBDEs, phtalates, PFAS
**Alteration of thyroid hormone–binding protein affinity**	PBDEs, PFAS, phtalates
**Disruption of thyroid hormone transport across cellular membranes**	BPA
**Alteration of deiodinase activity**	BPA, PBDEs,PFAS, phtalates
